# Automatic measurement of mesenteric vascular and portal vein parameters via PE-NET in the diagnosis of Crohn’s disease

**DOI:** 10.3389/fmed.2025.1634056

**Published:** 2025-10-23

**Authors:** Weize Xu, Liangfang Zheng, Kun Zhang, Bosheng He

**Affiliations:** ^1^Department of Radiology, Affiliated Hospital 2 of Nantong University, Nantong, Jiangsu, China; ^2^School of Electrical Engineering and Automation, Nantong University, Nantong, Jiangsu, China; ^3^Clinical and Translational Medicine Center, Affiliated Hospital 2 of Nantong University, Nantong, Jiangsu, China

**Keywords:** Crohn’s disease, PE-NET, deep learning, computed tomography enterography, mesenteric vascular, portal vein

## Abstract

**Objective:**

Vascular changes are concomitant of the course of Crohn’s disease (CD). In this study, we evaluated the value of the parallel encoder network (PE-NET) method for the automated measurement of mesenteric vascular and portal vein parameters and explored the performance of PE-NET combined with support vector machine (SVM) classifier in CD diagnosis.

**Methods:**

The automatic vascular segmentation model was trained using computed tomography enterography (CTE) imaging data from our hospital based on PE-NET. The segmentation performance of the trained model was evaluated using the sensitivity (SEN), the Dice Similarity Coefficient (DSC), and the Average Hausdorff Distance (AHD). Then, the model was used for the automatic measurement of vascular parameters in the classification set, and machine learning classifier SVM was applied based on selected vessel features. The diagnosis performance of the PE-NET + SVM model was evaluated and compared with that of human radiologists and the clinical biomarkers [C-reactive protein (CRP) and fecal calprotectin (FCP)]. The impact of PE-NET + SVM on the reading time of radiologists was also evaluated.

**Results:**

The segmentation dataset included the CTE data from 54 CD patients and 20 healthy controls. The classification dataset included the CTE data from 40 CD patients and 45 healthy controls. We found that PE-NET performed well in the vascular segmentation of the superior mesenteric artery (SMA), portal vein (PV), and abdominal aorta (AA) in both validation sets and the testing set. Vascular parameters were automatically extracted by PE-NET. We found that the mesenteric artery, portal vein, abdominal aorta, and the ratio of portal vein to superior mesenteric artery or abdominal aorta were increased in the testing set, with no statistical difference between the automatic measurement obtained using PE-NET and the manual evaluation of CTE. Moreover, an support vector machine (SVM) classifier was applied for CD diagnosis based on the vascular parameters. The F1 scores indicated the comparable diagnostic ability of PE-NET + SVM to senior radiologists with over 10 years of experience, and the receiver operating curves (ROCs) revealed that the area under the curve (AUC) of PE-NET + SVM was 0.934, which was higher than those of clinical biomarkers such as FCP (AUC of 0.913) and CRP (AUC of 0.893), suggesting the great potential of PE-NET in aiding CD diagnosis. Additionally, the reading time of a junior radiologist on CTE images was significantly reduced and comparable to that of a senior radiologist with the help of PE-NET.

**Conclusion:**

The PE-NET enables the automated measurement of mesenteric vascular and portal vein parameters and potentially assists the efficient diagnosis of CD.

## Introduction

Crohn’s disease (CD) is a chronic inflammatory disease of the gastrointestinal tract, with increasing incidence globally (1). It is more prevalent in patients younger than 30 years, and the course of this disease is progressive with relapsing attacks, along with intestinal complications such as fibrosis and colorectal cancer (1, 2). The early diagnosis is of vital significance for the effective treatment and outcomes of CD patients. Currently, although less invasive biomarkers such as C-reactive protein and Erythrocyte sedimentation rate (ESR) correlate with disease activity, the specificity remains a challenge. Consequently, the diagnosis of CD still relies on endoscopy and histological examinations, and the inherent limitations such as the invasiveness, the tolerance of patients, and the inability to examine segments proximal to the terminal ileum affect the accurate diagnosis of CD (3, 4). The delayed diagnosis often occurs due to the time lag between the onset of inflammation and the appearance of signs and symptoms, leading to an increased risk of adverse complications (5). Therefore, it is essential to develop novel methods for the early and effective diagnosis of CD.

Imaging examination is also an important part of CD diagnosis. Increasing evidence has shown that cross-sectional imaging techniques such as CT, MRI, and ultrasound serve as useful approaches for CD diagnosis. Although both endoscopic and imaging examinations can monitor the disease activity, the imaging techniques are also valuable in monitoring CD progression (6, 7). CT and MRI manifestations, such as mucosal hyperenhancement, wall thickening, and comb sign, have been revealed to be associated with active inflammation in CD (8). Moreover, the abnormalities in vascular alteration are associated with the inflammation and progression of CD (9). However, the evaluation of imaging features such as mesenteric and portal vascularity remains subjective, which makes it difficult to quantitatively compare these features with the severity of underlying inflammation and bowel injury.

In recent years, deep learning techniques have attracted increasing attention in the field of medical image analysis. Among the deep learning algorithms, the convolutional neural networks (CNNs) have performed well with excellent feature extraction and expression capabilities in computer vision and are widely used in classification, segmentation, object detection, and registration (10). The establishment of 2D and 3D U-Net has been widely used for the processing of medical images, and automatic segmentation algorithms based on these models have been used for organs, tissues, and vessels; however, the accuracy for small vessel segmentation remains unsatisfactory (11). PE-NET is a parallel encoder network suitable for the analysis of multimodal data in medical imaging, such as CT and MRI (12). Compared with the previous U-Net, which struggles in processing the large-scale change of vessels, the PE-NET algorithm with a self-adaptive feature shows great potential in the visualization of the whole vessel. Currently, the PE-NET has been applied for the segmentation of the inferior mesenteric artery in the abdomen (12, 13). However, the value of PE-NET in CD diagnosis remains largely unknown.

In this study, we aimed to investigate the performance of PE-NET in abdominal vascular segmentation and evaluate the value of PE-NET for the automated measurement of mesenteric vascular and portal vein parameters in the diagnosis of CD. The findings of this study might provide novel insights into the detection and management of CD.

## Materials and methods

### Study design

This retrospective study was conducted at our hospital. The PE-NET model, established as previously described, was applied in this study (12).

### Study subjects

To train the model, this study collected the imaging data and other relevant clinical data of 54 CD patients admitted to our hospital from January 2014 to December 2024 and 20 healthy control individuals without intestinal inflammation (validated by endoscopic examination). The training/validation set included 63 cases, and the testing set included 11 patients. The external validation dataset included 20 abdominal images of CD patients and 20 abdominal images of healthy controls obtained from the open public database.^1^ For the datasets used for the classification model, a total of 85 cases were included, with 30 cases in the training/validation set and 55 cases in the testing set. The study was approved by the Ethics Committee of our hospital and conforms to the principles of the Declaration of Helsinki. The inclusion criteria were as follows: (1) CD patients confirmed by histological examination, (2) those with CTE imaging data, (3) those who underwent endoscopic examination before or after imaging examination; and (4) those with comprehensive clinical data. The exclusion criteria were as follows: (1) patients who received abdominal surgery before the examination; (2) patients with scanning images of poor quality; and (3) patients with comorbidities such as cancer, coronary heart disease, and other gastrointestinal diseases.

### CT imaging

The CT scanning was conducted using a FORCE CT scanner (Siemens, Berlin, Germany). Patients were instructed to undergo standard bowel preparation before the CT examination, including a low-slag diet 2 days before the examination, a liquid diet 1 day before the examination, and fasting for 4–8 h before the examination. The scan area was from the top of the diaphragm to the lower margin of the pubis, and patients were in the supine position. The scan parameters were set as follows: tube voltage at A: 90 kV, B: Sn150 kV, tube current at A: 144 mass, B: 90 mAs, pitch of 1.0, speed of 0.5 s, collimation of 2 × 192 × 0.6 mm, slice thickness of 1 mm, and slice gap of 1 mm. Patients were first injected with 75 mL of iopromide (370 mgI/ml) at 3.5 mL/s in the antecubital vein and then with 20–30 mL of normal saline. The monitoring methods were applied based on a three-stage enhanced scanning automatic tracking technique. When the aortic monitoring threshold reached 100 HU, the arterial phase scan was triggered. The venous phase scan was performed 40 s later, followed by a delayed phase scan conducted 80 s after completion of the venous phase scan. The images were sent to a post-processing workstation (syngo via, VB20, Siemens Medical Solutions, Forchheim, Germany) for further processing.

### Processing and analysis of CT images

**Figure 1:**
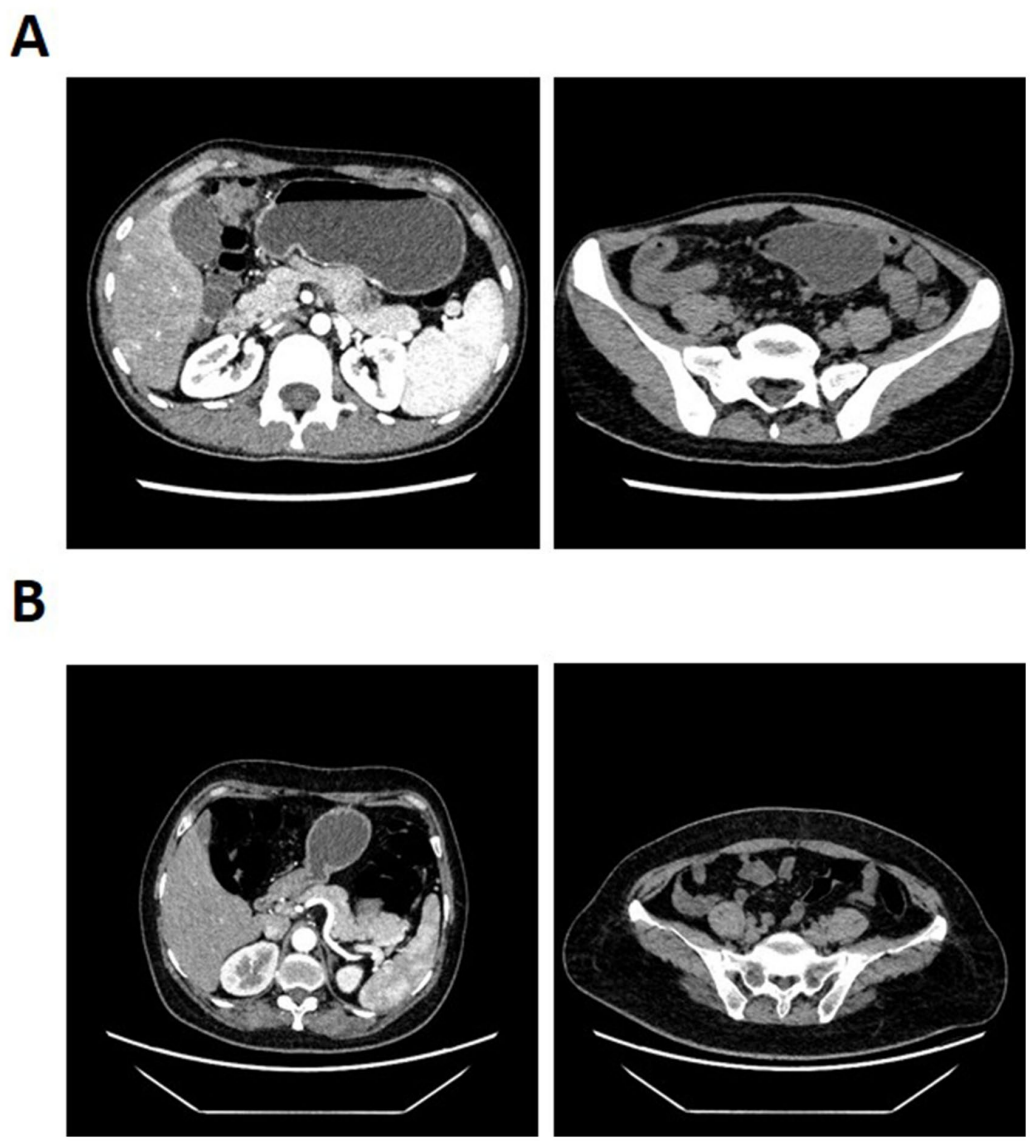
Computed tomography enterography (CTE) images from **(A)** a CD patient and **(B)** a healthy control individual.

The 120 kVP images with a slice thickness of 1 mm were obtained after inputting the raw data into the post-processing workstation. The virtual monoenergetic images with computational fusion were also obtained under the dual energy Mono+ mode (40–90 KeV) with the slice thickness of 1 mm. The cross-sectional images with a slice thickness of 3 mm and a slice gap of 3 mm were reconstructed for the above images. Two radiologists, each with over 10 years of experience and blinded to patient information, delineated the vessels. This was reviewed by a specialist with over 20 years of experience in abdominal imaging diagnosis. The robustness assessment was conducted in cases with anatomical variability or disease-induced deformation by evaluating the ability of the model in visualizing major vascular variants compared to the expected anatomical course based on CT images. The vessels delineated in consensus by radiologists were referred to as the ground truth. Figure 1 shows the example of CT images from a CD patient and a healthy control individual.

### PE-NET model

PE-NET was established as previously described (12, 13). It is a novel parallel network combining a transformer with CNN and is composed of a contracting path (encoder), an expanding path (decoder), and skip connections.

### Feature fusion and classification

The features of vessel parameters were fused and input into a support vector machine (SVM) for diagnosis, with the parameters of C = 1, gamma = ‘scale’, kernel = ‘rbf’, and degree = 3.

### Observational indicators

(1) To validate the performance of the model, voxel-based metrics including the sensitivity (SEN), Dice Similarity Coefficient (DSC), and Average Hausdorff Distance (AHD) were evaluated. Considering vascular connectivity, the evaluation of AHD is of top priority, followed by the DSC and SEN values.

(2) The vascular morphological characteristics, such as the diameter of superior and inferior mesenteric artery, the portal vein, and the abdominal aorta, were measured. The ratio of the portal vein/aorta or the portal vein/superior mesenteric artery diameter was evaluated to minimize the effect of patient size.

(3) Observer studies: Four radiologists of varying experience were included in the observation studies. The diagnostic performance of PE-NET + SVM was compared with that of human radiologists using F1 scores. The inter-rater consistency among human radiologists was evaluated using Cohen’s kappa scores.

(4) Traditional imaging indicators obtained from CT imaging were evaluated, including multisegmental bowel involvement, bowel wall thickening, mural stratification, mesenteric vessel engorgement, lymph node enlargement, and increased mesenteric fat density.

### Statistical analysis

SPSS 23.0 software and GraphPad Prism 8.0 software were used for data analysis. The Shapiro–Wilk test was used to evaluate the normal distribution of the data. For data that conformed to normal distribution, the measurement data were shown as the mean ± SD and evaluated using Student’s *t*-test. Categorical data were shown as the frequency and percentage (%), and comparisons between groups were conducted using the chi-square test. For measurement data that did not conform to normal distribution, data were shown as the median (Q25 and Q75) and compared with the Mann–Whitney *U* test. A *p*-value of <0.05 was regarded as a statistically significant difference.

## Results

### Clinicopathological features of CD patients in the segmentation set

This study included 54 CD patients and healthy control individuals. The mean age was 25.32 ± 4.36 for CD patients and 26.24 ± 3.96 for healthy controls. Among the CD patients, 17 were women and 37 were men. There were 8 women and 12 men in the healthy control group. There was no statistical significance between the baseline characteristics, including age and sex of CD patients and healthy controls (*p* > 0.05), while the CRP and ESR levels of CD patients were significantly higher than those of the healthy controls, and the BMI of CD patients was significantly lower relative to healthy controls (*p* < 0.05). Moreover, the CTE showed that, among CD patients, 26 had multisegmental bowel involvement, 31 had mural stratification, 40 showed comb signs, 12 had lymph node enlargement, 54 showed bowel wall thickening and mesenteric fat thickness of 0.85 ± 0.42, which were all significantly higher compared with the healthy controls (*p* < 0.05, Table 1).

**Table 1:** Baseline clinical characteristics of CD patients and control individuals in the segmentation cohort.

Variables	CD patients (*n* = 54)	Healthy controls (*n* = 20)	*P*
Age (years)	25.31 ± 4.35	26.20 ± 3.93	0.428
Sex			0.491
Female	17 (31.48)	8	
Male	37 (68.52)	12	
BMI (kg/m^2^)	20.85 ± 1.27	22.20 ± 3.27	0.013
Location			
L1 (ileum)	12 (22.22)	–	
L2 (colon)	0 (0)	–	
L3 (ileocolon)	42 (77.78)	–	
Disease activity			
Mild	18 (33.33)	–	
Moderate–severe	36 (66.67)	–	
Abdominal pain	31 (57.41)	–	
Diarrhea	29 (53.70)	–	
Fever	6 (11.11)	–	
Weight loss	16 (29.63)	–	
Bloody stools	8 (14.81)	–	
FCP (μg/g)	789 (383.7,1,052)	24.76 (21.3,33.05)	<0.001
CRP (mg/L)	13.86 (11.36,18.33)	2.75 (1.86, 4.50)	<0.001
ESR (mm/h)	48 (27,59)	6.5 (4, 14.5)	<0.001
CT features			
Multisegmental bowel involvement	26	0	<0.001
Mural stratification	31	0	<0.001
Comb sign	40	0	<0.001
Lymph node enlargement (>1 cm in short diameter)	12	0	0.021
Bowel wall thickening (>3 mm)	54	5	<0.001
Mesenteric Fat Thickness (cm)	0.85 ± 0.42	0.61 ± 0.23	0.018

CD, Crohn’s disease; FCP, fecal calprotectin; CRP, C-reactive protein; ESR, erythrocyte sedimentation rate.

### Assessment of the segmentation performance of PE-NET

Data from 74 participants (54 CD patients and 20 healthy controls) were used for modeling, of which data from 63 cases were used for the training and validation sets and those from 11 cases were used for the testing set. The automated segmentation performance of the model was evaluated with the five-fold cross-validation and compared against the ground truth expert-labeled segmentations. The data of 20 CD cases and 20 healthy controls from a public dataset were used for external validation. The segmentation performance of PE-NET of each type of vessel (SMA, PV, and AA) was tested and evaluated as shown in Table 2. The model showed comparable SEN, DSC, and AHD across the three cohorts, although the SEN and DSC values in the external validation group were slightly slower, and the AHD were slightly higher for PV segmentation compared with the internal validation group.

**Table 2:** The segmentation performance in SMA, PV, and AA across cohorts.

Vessels	SEN (%)			Dice (%)			AHD		
	Internal validation set (*n* = 10)	Testing set (*n* = 11)	External validation set (*n* = 40)	Internal validation set (*n* = 10)	Testing set (*n* = 11)	External validation set (*n* = 40)	Internal validation set (*n* = 10)	Testing set (*n* = 11)	External validation set (*n* = 40)
SMA	93.23 ± 6.41	91.88 ± 5.49	92.72 ± 6.43	92.88 ± 5.32	92.24 ± 5.61	90.39 ± 5.74	5.33 ± 4.45	5.58 ± 4.52	5.22 ± 4.59
PV	91.83 ± 4.58	89.68 ± 5.63	89.21 ± 4.97	89.29 ± 4.70	87.48 ± 4.59	85.29 ± 6.18	6.19 ± 4.56	6.67 ± 4.55	7.21 ± 5.60
AA	90.04 ± 5.25	88.62 ± 6.94	88.75 ± 5.77	87.60 ± 4.81	85.91 ± 5.07	85.14 ± 5.16	7.36 ± 4.82	7.74 ± 5.27	7.33 ± 5.41

SMA, superior mesenteric artery; PV, portal vein; AA, abdominal aorta; AHD, Average Hausdorff Distance.

### Measurement of mesenteric vascular and portal vein parameters

**Figure 2:**
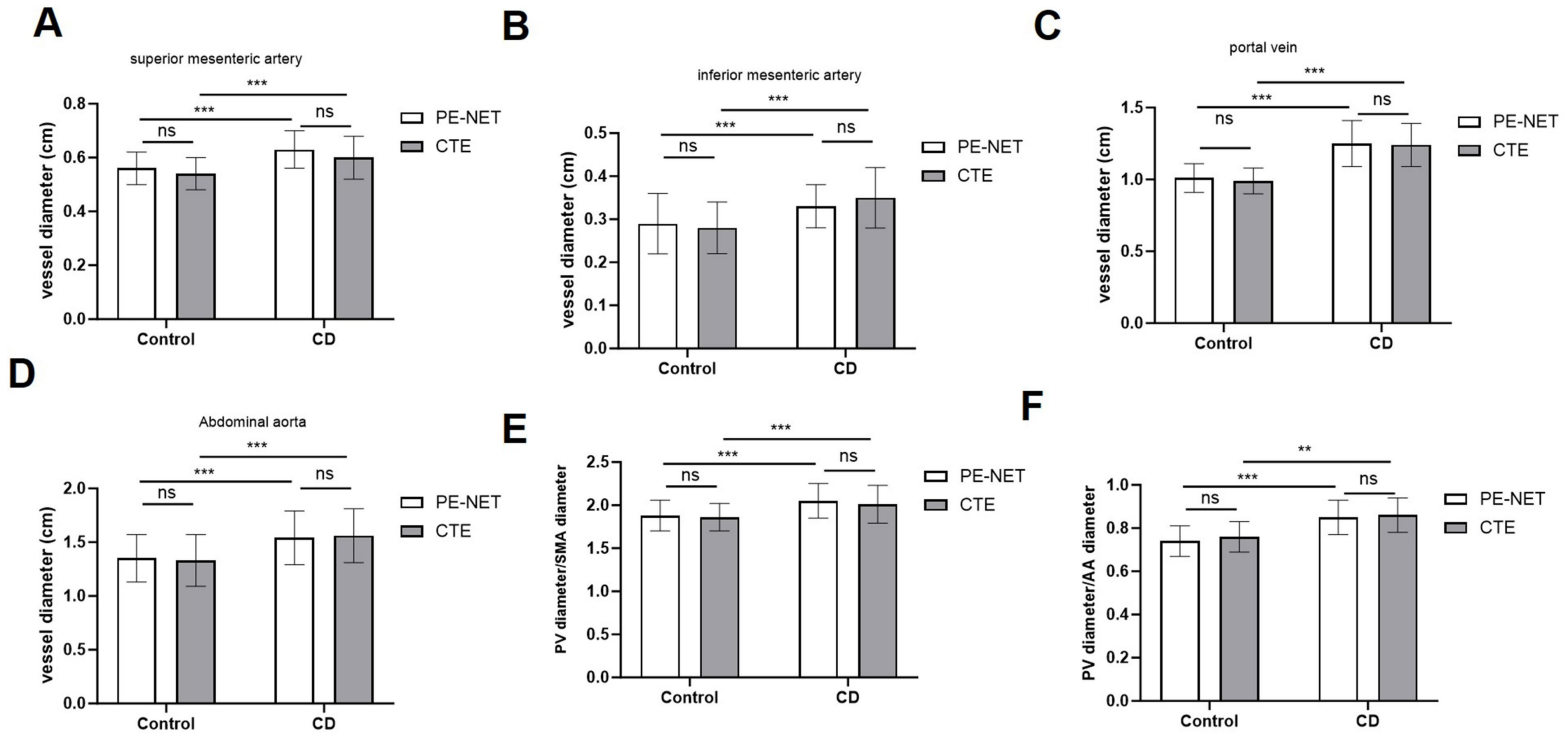
The measurement accuracy of mesenteric vascular and portal vein parameters by PE-NET. The diameter of **(A)** superior mesenteric artery (SMA), **(B)** inferior mesenteric artery (IMA), **(C)** portal vein (PV), and **(D)** abdominal aorta (AA), as well as **(E)** the ratio of PV/SMA and **(F)** PV/AA in control and CD patients in the testing set were assessed via PE-NET automatic measurement or CTE evaluation. Ns, non-significant; *p < 0.05, **p < 0.01, ***p < 0.001.

The accuracy of the automatic segmentation by PE-NET was evaluated in the testing set. As measured by PE-NET or CTE evaluation, the diameter of the superior mesenteric artery, the inferior mesenteric artery (IMA), the portal vein, and the abdominal aorta (AA), as well as the ratio of portal vein (PV)/superior mesenteric artery (SMA) and PV/AA diameter was significantly increased in CD patients compared with the control group (Figures 2A–F). Moreover, we found that the bias of mesenteric vascular, portal vein, and abdominal aorta parameters between PE-NET and CTE evaluation showed no significant difference, which indicated minimal systemic error. Overall, these data revealed that PE-NET performed well in the automated measurement of mesenteric vascular and portal vein parameters as well as in the calculation of PV/SMA and PV/AA diameter.

For the evaluation of vascular variants (Table 3), the successful visualization rate of the model was 50% (1 of 2) and 66.67% (2 of 3) in the internal validation and testing groups, respectively.

**Table 3:** Detection of vascular variations based on PE-NET.

Dataset	Variations	Detection rate (%)
Internal validation set	SMA → RHA (N = 2)	1 (50%)
Testing set	SMA → RHA (N = 1)	2 (66.67%)
	SMA → CHA (N = 2)	

SMA, superior mesenteric artery; CHA, common hepatic artery; RHA, right hepatic artery.

### Assessment of classification results based on SVM machine learning method

**Figure 3:**
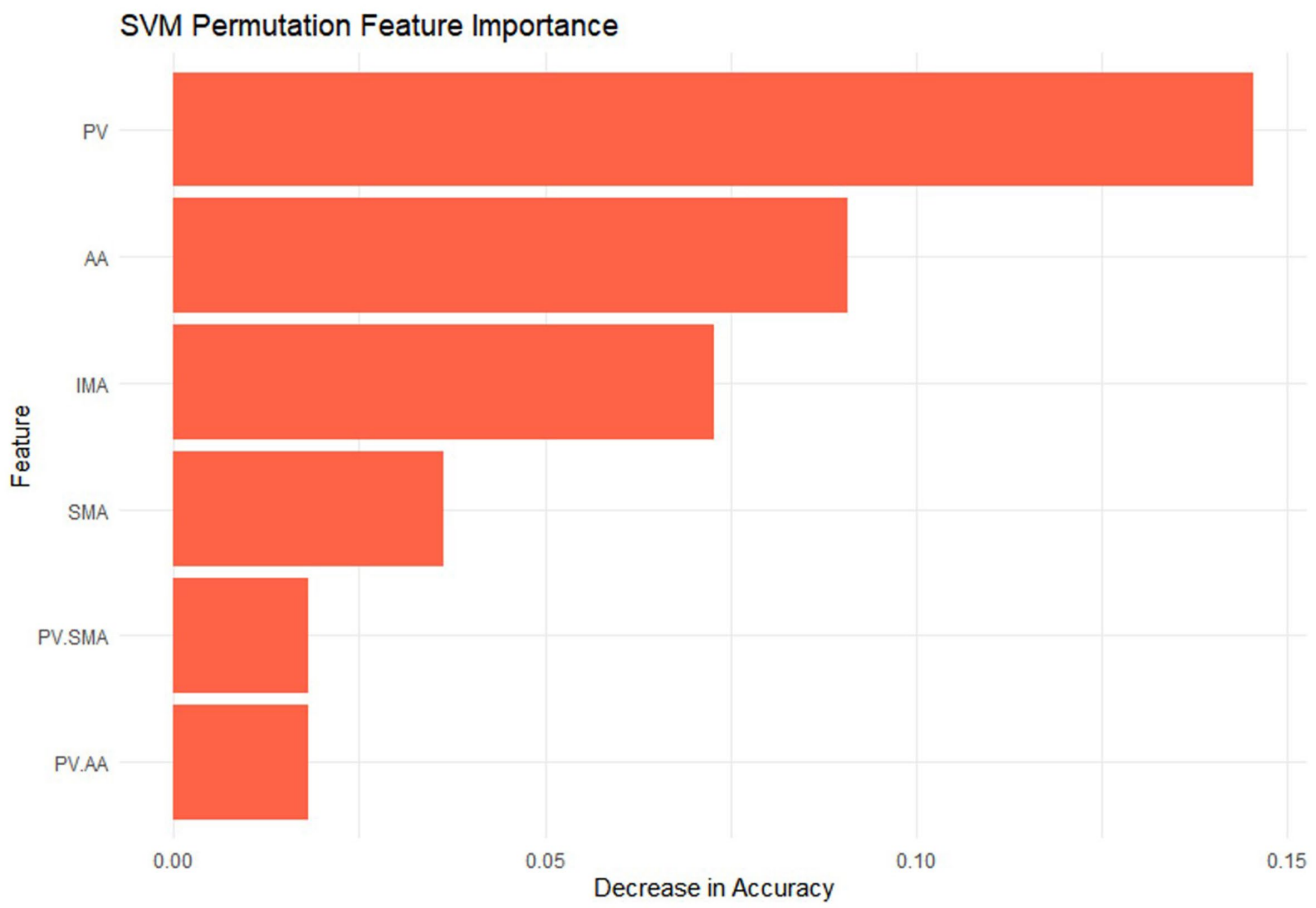
The permutation feature importance in the SVM classifier.

The SVM classifier was applied based on the vessel parameters automatically measured by PE-NET in the classification set. The classifying accuracy reached 81.82% (Sensitivity: 75.86%; Specificity: 88.46%), and the AUC value of the SVM model was 0.934 in the testing set (Table 4). Figure 3 shows the importance of the permutation feature for the SVM model and highlights the contribution of the selected features to the diagnostic efficiency. The results indicated the top importance of the PV diameter to the SVM model.

**Table 4:** Classification performance of the SVM machine learning model.

Dataset	AUC	Accuracy (%)	SEN (%)	SPE (%)
Training set	0.955	85.45	86.96	84.38
Validation set	0.881	80	71.43	81.48
Testing set	0.934	81.82	75.86	88.46

### Comparison between PE-NET + SVM and human radiologists or clinical biomarkers in diagnosis performance

The diagnosis performance of the PE-NET + SVM model alone was compared with that of human radiologists with varying expertise using F1 scores. The results showed that the model exhibited better performance compared with junior human radiologists with over 5 years of experience and nearly comparable performance to the senior human radiologists with over 10 years of experience (Table 5). When combined with the PE-NET + SVM model, the junior human radiologists showed improved F1 scores that were comparable to the senior human radiologists without PE-NET + SVM model assistance. We also found that the F1 scores of senior human radiologists were increased with the aid of the PE-NET + SVM model, suggesting the model as a promising tool in aiding the clinical diagnosis of radiologists. Additionally, the human expert variability was evaluated by Cohen’s kappa scores, and inconsistencies were observed between the junior and senior radiologists. The results suggested that applying the PE-NET + SVM model might exert improved stability in clinical diagnosis (Table 6).

**Table 5:** F1 scores of the PE-NET + SVM model and human radiologists in CD diagnosis.

Participant	Years of experience	F1 score
Radiologist 1	5	0.735
Radiologist 1 + model	–	0.863
Radiologist 2	5	0.724
Radiologist 2 + model	–	0.846
Radiologist 3	10	0.844
Radiologist 3 + model	–	0.923
Radiologist 4	10	0.84
Radiologist 4 + model	–	0.941
Model	NA	0.815

**Table 6:** Inter-rater consistency of four human radiologists.

Rater (row)	Radiologist 1	Radiologist 2	Radiologist 3	Radiologist 4
Radiologist 1	–	0.420	0.235	0.227
Radiologist 2	–	–	0.237	0.238
Radiologist 3	–	–	–	0.854
Radiologist 4	–	–	–	–

**Figure 4:**
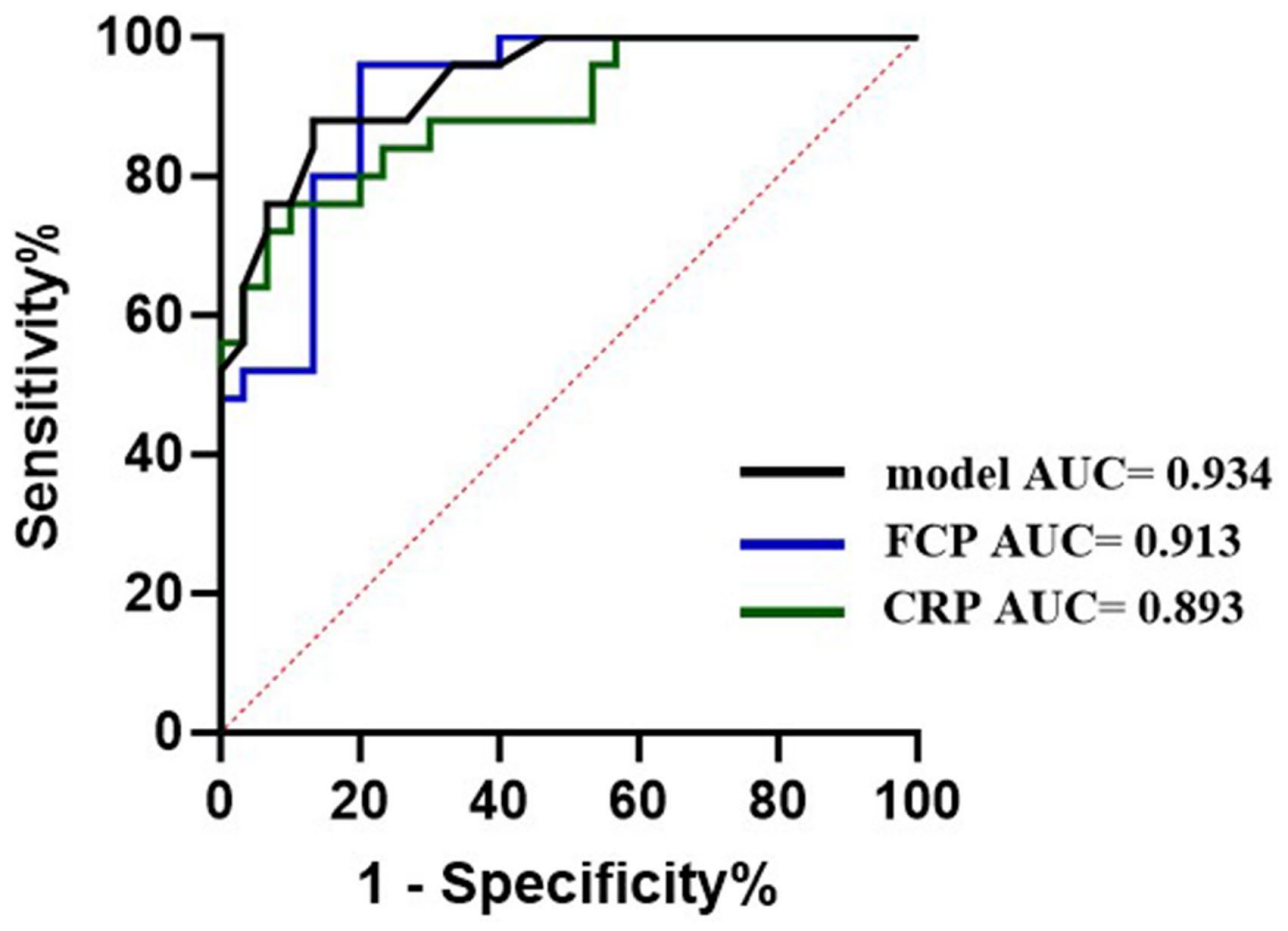
The diagnostic ability of PE-NET compared with blood and fecal biomarkers on CD.

Moreover, we also compared the diagnostic ability of the PE-NET + SVM model with two clinical biomarkers (FCP and CRP) of CD. The results of the ROC analysis showed that the AUC value was 0.934 for the model, 0.913 for FCP, and 0.893 for CRP in the testing set, which indicated the good performance of the PE-NET + SVM model in CD diagnosis (Figure 4).

### The effects of PE-NET + SVM model on the diagnostic efficiency of CD patients

Moreover, we evaluated the potential of PE-NET in improving the diagnostic efficiency of CD. The results showed that the average reading time and total reading time for a junior radiologist (radiologist 1) were significantly reduced with the aid of PE-NET + SVM model and comparable to that of a senior radiologist (radiologist 3), while the average reading time for a senior radiologist (radiologist 3) was not evidently decreased by the application of PE-NET + SVM. The results suggested that the diagnostic efficiency of junior radiologists was improved with the help of the PE-NET + SVM model. Though the reading time was not significantly decreased for a senior radiologist, the application of the model might potentially reduce their burden in correcting the mistakes of junior radiologists and decrease the misdiagnosis or missed diagnosis in the clinic (Table 7).

**Table 7:** Effects of PE-NET + SVM model on the diagnostic efficiency of radiologists.

Measurement	Radiologist 1	Radiologist 1 + model	Radiologist 3	Radiologist 3 + model	*P^1^*	*P^2^*	*P^3^*
Time (min)	5.9 ± 1.1	4.4 ± 0.8	4.2 ± 1.2	4.1 ± 1.1	<0.001	0.981	<0.001
Total time	321.8	239.3	230.8	226.6			

P1 indicates the statistical difference between the Radiologist 1 and Radiologist 1 + model. P2 indicates the statistical difference between Radiologist 3 and Radiologist 3 + model. P3 indicates the statistical difference between Radiologist 1 + model and Radiologist 3.

## Discussion

Crohn’s disease (CD) is a lifelong chronic intestinal inflammatory disease, with an incidence peak between the second and fourth decades of life, significantly affecting the quality of life of patients (1, 14, 15). Early diagnosis is essential for the determination of long-term therapeutic plans. Endoscopic and histological evaluation is essential for the confirmation of CD, while the application is limited by the inability to observe the bowel wall architecture, the subjective interpretation, and the low tolerance of patients (16). The non-invasive diagnostic methods such as cross-sectional imaging show higher tolerance and are valuable not only for the early examination but also for monitoring of the disease progression (6, 17). In this study, we developed a PE-NET model for automated measurement of mesenteric vascular and portal vein parameters, and the combination with SVM classifier enables the discrimination of CD disease activity. The segmentation and classification performance of the PE-NET + SVM was validated, and the diagnostic efficiency of CD was improved with the help of PE-NET + SVM.

Accumulating evidence has shown that vascular changes, including angiogenesis and lymphangiogenesis are crucially involved in CD progression (18–21). Notably, the enlarged artery or portal vein vessels, which have been observed in CD patients, are likely related to the increasing requirement of oxygen and nutrients of the affected segment in the course of CD (22). For example, the mesenteric components, such as the vascular system, are involved in the gut dysbiosis-adaptive immunity-mesentery-body axis and are critically associated with the CD progression (9). The mesenteric blood flow based on MRI has been linked with intestinal inflammation in CD patients, with a positive correlation between the blood flow and C-reactive protein and fecal calprotectin (23). A study by Yekeler et al. (24) has also revealed that the mean diameter and flow volume of the superior mesenteric artery (SMA) are increased in active CD patients than in inactive CD patients. A study has evaluated the difference in splanchnic vascular flow by measuring the transverse diameters of the main portal vein and abdominal aorta and found elevated diameters in the portal vein and portal vein/aorta ratio in CD patients (22). Therefore, exploring the alteration of abdominal vessels such as the SMA and portal vein can potentially contribute to the diagnosis of CD.

In recent years, deep learning techniques-based segmentation methods have boasted the automatic processing of CT images for detection and classification tasks (25–28). However, it remains a challenging task to automatically segment abdominal vessels because of the multi-scale nature of vessels, blurred boundaries, low contrast, and vascular cracks in Maximum Intensity Projection (MIP) images. In this study, the PE-NET method is used for the automatic segmentation of abdominal vessels, including the SMA, the portal vein, and the abdominal aorta. This network applied a new network architecture CGS that integrates redundant features in space into the target vessel to obtain the maximum coronal vessel (13). Compared with other deep learning models, such as 3D U-net, AU, and CAS models in vessel segmentation, PE-NET shows higher sensitivity and lower AHD (12). Our model performs well in capturing the overall vascular structure, especially in learning vascular edge features, while the 3D U-net, AU, and CAS models showed under-segmentation in tiny vessels, and extensive vascular disconnections at varying degrees (12). Compared with the segmentation by junior radiologists, the PE-NET showed improved performance with relatively higher F1 scores, suggesting the usability of this method in abdominal vessel segmentation. Moreover, the automatic measurement of mesenteric vascular and portal vein parameters showed no significant difference with the ground-truth measurement results, and the results indicated the increase in diameter of SMA, IMA, PV, and AA, as well as the ratio of PV/SMA and PV/AA, which are consistent with the previous findings (22).

Accurate and efficient assessment of disease is of vital importance for the monitoring and treatment of CD. In our study, we revealed the ability of PE-NET with an SVM classifier in the diagnosis of CD patients. Compared with blood and fecal biomarkers, the PE-NET with SVM classifier showed a relatively higher AUC and increased specificity, suggesting great potential in clinical CD diagnosis. Additionally, the reading time of CT images for a junior radiologist was significantly decreased with the help of PE-NET + SVM and was comparable to that of a senior radiologist. Although the reading time of a senior radiologist was not significantly improved by PE-NET + SVM, their burden of correcting the junior radiologists can possibly be eased.

Despite the advantage of the PE-NET model, this study also possesses some limitations. First, the sample size of the dataset used in this study was relatively small, which potentially caused model overfitting and influenced the generalizability of the model. Second, the CT images used for generating the training and internal validation set were obtained from a single institution using a Siemens FORCE CT scanner. While the five-fold cross-validation was used to improve the model robustness and external validation was conducted using imaging data from an open-source database, future research is warranted to include more imaging modalities and data from multiple centers to enhance the generalizability and validate the performance as well as clinical applicability of the model across different scanners, acquisition parameters, or institutions.

In conclusion, this study applied the PE-NET method for the automatic evaluation of mesenteric vascular and portal vein parameters, with good performance in vascular segmentation, demonstrated great performance in CD diagnosis compared with blood and fecal biomarkers, with high sensitivity and specificity, and improved the diagnostic efficiency of radiologists. Given the potential of PE-NET in CD diagnosis, the findings of this study might provide novel insight into the CD detection and management in the future.

## Data Availability

The datasets presented in this study can be found in online repositories. The names of the repository/repositories and accession number(s) can be found in the article/supplementary material.

## Footnotes

^1^ https://crohnipi.ls2n.fr/en/crohn-ipi-project/
